# The Effect of Dietary Zinc Oxide Nanoparticles on Growth Performance, Zinc in Tissues, and Immune Response in the Rare Minnow (*Gobiocypris rarus*)

**DOI:** 10.1155/anu/9553278

**Published:** 2024-12-02

**Authors:** Huanhuan Li, Menghan Wu, Jinming Wu, Jing Wan, Yongfeng He, Yifan Ding, Jun Liu, Liangxia Su

**Affiliations:** ^1^Hubei Key Laboratory of Animal Nutrition and Feed Science, Engineering Research Center of Feed Protein Resources on Agricultural By-Products, Ministry of Education, Wuhan Polytechnic University, Wuhan, China; ^2^The Key Laboratory of Aquatic Biodiversity and Conservation of Chinese Academy of Sciences, Institute of Hydrobiology, Chinese Academy of Sciences, Wuhan, China; ^3^University of Chinese Academy of Sciences, Beijing, China; ^4^Key Laboratory of Freshwater Biodiversity Conservation, Ministry of Agriculture and Rural Affairs of China, Chinese Academy of Fishery Sciences Yangtze River, Fisheries Research Institute, Wuhan, China

**Keywords:** *Gobiocypris rarus*, growth, immune function, the number of erythrocytes and leukocytes, ZnO NPs

## Abstract

In recent years, zinc oxide nanoparticles (ZnO NPs) have gained attention as feed additives due to their high bioavailability. However, research on their impact on fish growth and health is limited. To investigate the influences of dietary addition of ZnO NPs on growth performance and immune function of rare minnow, rare minnows were fed diets with different ZnO NPs content. Growth analysis showed that ZnO NPs had a negative effect on the weight of rare minnow, decreasing and then increasing condition factors (CFs) and specific growth rate. Additionally, the accumulated zinc (Zn) level was significantly higher (*p*  < 0.05), and the liver injury index was significantly higher (*p*  < 0.05) in the dietary ZnO NPs group compared to the control group. The number of erythrocytes and leukocytes in blood samples increased and then decreased after treatment with ZnO NPs. It was further found that ZnO NPs as a dietary supplement significantly increased the Zn content and markedly repressed the expression of growth-related genes after 60 days of accumulation in muscle tissues, and accumulation in liver tissues for 60 days significantly enhanced the expression of immune modulation–related genes expression (*p* < 0.05). The findings suggested that short-term supplementation of ZnO NPs could positively affect fish growth and immune function. However, prolonged supplementation of dietary ZnO NPs resulted in reduced body weight and compromised immune function owing to the buildup of Zn in different tissues.

## 1. Introduction

Fish is an important component of human diet, providing high-quality protein and critical elements. Micronutrients play an important role in fish in promoting growth, health, reproduction, immune system, and disease resistance [1]. Fish are among the vertebrates that depend on zinc (Zn) for proper development and metabolism [2, 3]. Fish species get their essential minerals and vitamins from water and feed [4]. The concentration of Zn in freshwater is not sufficient to come across the requirements of fish [5, 6]. It is, therefore, recommended that Zn be added to aquafeeds that meet their requirements for optimal performance.

Zinc oxide (ZnO) is widely recognized as a crucial Zn supplement in farmed animals' diets [7]. Recently, nanoscale particles of trace minerals have been become an alternative to conventional mineral supplements. In comparison to bulk minerals, nanoscale particles offer novel properties, such as small size, high bioavailability, wide surface area, excellent adsorption capacity, strong catalysis, and require lower dosage [8]. They also improve growth and immunity by providing antioxidant and antibacterial benefits [9, 10]. As for a source of Zn, zinc oxide nanoparticles (ZnO NPs), an important nanomaterial, have attracted much attention in recent years for their application in agriculture and animal feed [11]. ZnO NPs, as a trace element supplement, are believed to increase the growth rate of fish, enhance their immunity, and have a positive effect on muscle tissue growth [12].

Although the U.S. Food and Drug Administration classifies this substance as “generally recognized as safe”, there are numerous reports indicating potential hazards to human health and the environment [13], as well as dietary toxicity in fish [14]. Studies have demonstrated the toxic impacts of ZnO NPs on aquatic species, involving cytotoxicity and genotoxicity [15], embryotoxicity [16], and alterations in transcriptional [17] or endocrine profiles at both cellular and molecular levels [18]. Exposure to ZnO NPs significantly decreased the frequency of *Cetobacterium* in the intestine while increasing the prevalence of several pathogens, resulting in the diminishment of pathways related to carbohydrate metabolism, translation, and replication and repair [19]. However, studies investigating the impact of ZnO NPs on the growth and health of fish species remain limited.

The rare minnow (*Gobiocypris rarus*) is a tiny freshwater fish native to China. Previous studies have shown that excess Zn causes growth and developmental effects in the embryos of rare minnow [20]. Consequently, the aim of this study was to evaluate in detail the effects of adding several levels of ZnO NPs to feed rations on the growth and development and liver immune function of rare minnow, with a view to providing a scientific basis for its application in feed additives.

## 2. Materials and Methods

### 2.1. Experimental Animals and Culture

In this research, 360 healthy mature rare minnows (0.35 ± 0.06 g per fish, 3 months old) were sourced from the National Aquatic Biological Resource Center. These minnows were acclimated for 1 week in eight-liter seamless glass tanks, and no mortality observed were observed. During both the acclimatization and experimental periods, a consistent 12-h light–dark photoperiod (08:00–20:00) was maintained. Water quality was rigorously monitored, with each 6-L tank being refreshed twice daily to ensure optimal conditions, to maintain temperature (25–27°C), pH (7.0–8.5), and dissolved oxygen content (7–9 mg/L). The minnows were provided a basal diet twice daily until satiety, with the ingredient composition detailed in Table S1 of the Supporting Information. All experiments received approval from the Ethics Committee of the Wuhan Polytechnic University (approval number WPUF20221008, approval date 8 October 2022).

### 2.2. Experimental Design

Commercial bare ZnO NPs (99.9% metals basis) used in the present study were purchased as a powder (MACKLIN Biochemical Technology Co., Shanghai, China) and the particle size measuring 30 ± 10 nm. Based on the results of previous studies on sub-chronic toxicity in 7 days larvae (unpublished) and Shukry et al. [21], 0, 20, and 60 mg/kg of ZnO nanoparticles were used as the dosage in this experiment. After 1 week of acclimatization, 360 healthy rare minnows (body weight: 0.35 ± 0.06 g, body length: 28.99 ± 0.05 mm) were randomized into three groups: the control group (basal diet + 0 mg/kg ZnO NPs), the N-20 group (basal diet + 20 mg/kg ZnO NPs), and the N-60 group (basal diet + 60 mg/kg ZnO NPs). Each group was housed in eight seamless glass tanks (*n* = 15 per tank). The experimental diets were supplemented with ZnO NPs during batching and mixing prior to pelleting and drying. The experimental period lasted for 60 days. Following feeding durations of 15, 30, 45, and 60 days, 30 fish from each cohort were euthanized using a solution of 200 mg/L MS-222, and their whole body, dorsal muscle, and liver were placed in liquid nitrogen for rapid freezing and then preserved at −80°C for later testing. Before dissection, the body weight and length of five rare minnows in each group were measured, and growth indicators were calculated as follows:

  $$\begin{array}{c} \text{Condition factor }\left(\text{CF},\%\right)=100\times W/L^{3} \end{array}$$

  $$\begin{array}{c} \text{Specific growth rate }\left(\text{SGR},\%/\text{day}\right)=100\times \left(\ln\;W_{t2}-\ln\;W_{t1}\right)/\left(t_{2}-t_{1}\right), \end{array}$$

where *W* is the body weight (g), *L* is the body length (mm), *W*_*t*2_ and *W*_*t*1_ are the body weight of the fish at *t*_2_ and the body weight of the fish at *t*_1_ in grams, *t*_1_ is the time (day) at which the samples were sampled, and *t*_2_ is the time (day) at which the samples will be collected at the next stage, with a 15 days interval between *t*_1_ and *t*_2_.

Their blood was taken for subsequent experiments. Each treatment was repeated three times. Feeding was suspended 12 h before sampling. During the acclimatization and experimental periods, water quality parameters were consistently controlled at a temperature of 25.8 ± 0.95°C, a pH level of 8.32 ± 0.21, and a dissolved oxygen concentration of 8.29 ± 0.52 mg/L. The nutritional composition of the feeds for each experimental group is given in Table S2 in the Supporting Information. The crude protein, ether extract, ash, and moisture in the experimental diets were analyzed in accordance with the standards GB/T 6435-2014, GB/T 6432-2018, GB/T 6433-2006, and GB/T 6438-2007, respectively.

### 2.3. Analysis of the Nutritional Levels of Rare Minnow

Crude protein, ether extract, ash, and moisture of pooled total body samples of three rare minnows in each group were measured following to GB/T 6435-2014, GB/T 6432-2018, GB/T 6433-2006, and GB/T 6438-2007. Each experiment was performed in triplicate, and the mean and standard deviation (SD) are presented.

### 2.4. Analyses of Zn Content in Muscle Tissues of Rare Minnow and Experimental Diets

The Zn concentration in muscle tissues of the rare minnow and experimental diets was determined utilizing inductively coupled plasma mass spectrometry (ICP-MS) (PerkinElmer NexlON 300X; Pekin Elmer, Waltham, MA, USA). Two muscle samples of rare minnow and 0.05 g feed ration from each group were digested separately in a microwave digestion system with 200 µL of HNO_3_ and incubated at 65°C for 24 h. A Following the digestion process, the addition of 9.8 mL of deionized water achieved a total volume of 10 mL, resulting in a 2% concentration of HNO_3_. Subsequently, the samples underwent analysis utilizing HPLC-ICP-MS. The sensitivity was 0.01 µg/g dry weight, with recoveries ranged from 96% to 98%. Zn concentrations in muscle of rare minnow and experimental diets were expressed as µg/g dry weight. Muscle samples and feed samples were subjected to three replications each, and the outcomes were reported as the average values accompanied by their respective standard deviations.

### 2.5. Analyses of Hematology

Immediately after euthanasia of two rare minnows in per group, blood samples (20 µL) were taken with a pipette through an oblique incision between the anal and caudal flippers. Add 1.99 mL of erythrocyte diluent (Haling, Shanghai, China) to the test tube, aspirate 10 µL of anticoagulated blood with a clean and dry micropipette, wipe off the remaining blood from the outside of the tube and add it to the bottom of the erythrocyte diluent, and then gently aspirate the upper layer of the clear liquid to clean the pipette twice, and then mix it immediately. The erythrocyte suspension was washed into the cell counter plate with a clean micropipette and counted using a Beckman–Coulter Z2 cell counter (USA). Add 1.99 mL of leukocyte diluent (Haling, Shanghai, China) to the test tube, aspirate 10 µL of anticoagulated blood with a clean and dry micropipette, wipe off the remaining blood from the outside of the tube and add it to the bottom of the leukocyte diluent, and then gently aspirate the upper layer of the supernatant to wash the pipette twice, and mix it immediately. After complete destruction of erythrocytes and remixing, the leukocyte suspension was washed into the cell counter plate with a clean micropipette and counted using a Beckman–Coulter Z2 cell counter (USA). Each experiment was performed in triplicate, and the mean and SD are indicated.

### 2.6. Assessment of Hepatic Injury

Paraformaldehyde-fixed hepatic samples were paraffin-embedded to visualize hepatic damage and stained with hematoxylin and eosin (H&E). Each group consisted of three liver tissue samples that were dissected and then fixed in paraformaldehyde at 4°C for 24 h. After fixation, the liver samples were graded, dehydrated in an ethanol series, and embedded in paraffin following standard histological procedures. Thick sections (5 µm) were cut from the paraffin blocks using a rotary microtome and stained with H&E. The sections were examined utilizing an Olympus CX33 light microscope (Olympus Corporation, Tokyo, Japan) equipped with a camera. The staining of the sections was performed by Wuhan Service Bio Technology Co., Ltd. (Wuhan, China).

To compare the degree of hepatic tissue injury caused by ZnO NPs, the liver injury status index (*I*_*h*_) was estimated according to the weighted index method proposed by Bernet et al. [22] for fish with modifications. All experiments were analyzed in three replicates. This method accounts for the biological significance (weights) of each observed alteration and its degree of spread (score). Weight values ranged between 1 and 3 (most severe), and scores ranged from 0 (no features/alterations observed) to 6 (diffuse). The liver injury status index was calculated using the following formula:

  $$\begin{array}{c} I_{h}=\sum_{1}^{j}w_{j}\times a_{jh}, \end{array}$$

where *I*_*h*_ is the liver injury status index for the individual *h*; *w*_*j*_ is the weight of the *j*th liver injury status alteration, and *a*_*jh*_ the score attributed to the *h*th individual for the *j*th alteration.

### 2.7. Analysis of Quantitative Real-Time PCR (qRT-PCR)

Total RNA was extracted from rare minnow muscle and liver samples separately (*n* = 3 replicates per group, 3 muscle or liver samples per replicate). Homogenization was performed in 5 mL of Trizol reagent (Invitrogen, Carlsbad, CA, USA), and RNA was then extracted. The quality of the isolated RNA was determined by analyzing the optical density at 260 and 280 nm using a spectrophotometer (Thermo Fisher Scientific, Waltham, MA, USA). Subsequently, 1000 ng of total RNA was back transcribed into cDNA utilizing Moloney Murine Leukemia Virus (M-MuLV) reverse transcriptase (Sangon Biotech, Shanghai, China) and oligo d(T) primers, resulting in a total reaction system of 20 µL. The synthesized cDNAs were frozen at −20°C for real-time polymerase chain reaction (RT-PCR) assay.

The RT-PCR analysis was performed using a SYBR Green PCR master mix from Bimake (Houston, TX, USA) on a fluorescence quantitative PCR instrument from Bio-Rad (California, USA). Each reaction solution includes 10 µL SYBR Green PCR master mix, 7 µL nuclease-free water, 2 µL cDNA (100 ng), 0.5 µL forward primer, and 0.5 µL reverse primer. The PCR protocol included an initial denaturation at 95°C for 5 min, preceded by 40 cycles of 95°C denaturalization for 10 s, 60°C anneal for 10 s, and 72°C elongation for 30 s. Subsequent disassociation curve evaluation was used to confirm single-product accumulation at the completion of every PCR run. All experiments were performed in triplicate. The reference gene *β*-actin was employed as an internal control to standardize the expression levels of the test genes, which were evaluated by the comparative C(T) method (2^−*ΔΔ*Ct^ method). RT-PCR primer sets for mRNA of growth-related genes (*gh*, *smt*, *mstn*, *igf1*, *igfbp5b*, *igfbp2a*, *igfbp3*, and *ghrb*) in muscle and immune-related genes (*tlr3*, *il-6*, *ifn-2*, *il-8*, *nf-кb*, and *myd88*) in liver are shown in Table S3, upporting Information. The qRT-PCR primer amplification efficiency isdetermined according to the approach of Zhao et al. [23].

### 2.8. Statistical Analysis

Statistical analysis was conducted utilizing IBM SPSS Statistics 27.0.1 (Armonk, NY, USA). The normality and variance equality were assessed using the Kolmogorov–Smirnov and Levene tests, respectively. With the fulfillment of the normality and homogeneity assumptions, the data were analyzed through one-way analysis of variance with Tukey's HSD post hoc test for repeated comparisons. The statistical value of the data was set at *p* < 0.05. The results were reported as mean ± SD. Graphs were generated utilizing GraphPad Prism version 7.0 (GraphPad Software Inc., La Jolla, USA).

## 3. Results

### 3.1. Growth Performance of Rare Minnow

To examine the impact of dietary ZnO NPs on the growth performance of rare minnow, the researchers measured the growth parameters in the rare minnow (Table 1). Supplementation of the diets with ZnO NPs had a negative effect on body weight. In comparison with the control group, the body length of the treated group increased at 15 days and decreased at the later stage. And condition factors (CFs) in the experimental group reduced in the early 30 days and improved at the later stage, but none of the changes were remarkable (*p* > 0.05). However, the specific growth rate (SGR) of the treated group showed a trend of significant decrease followed by a remarkable increase compared to the control group (*p* < 0.05). Specifically, at 45 days, the SGR of N-20 and N-60 were 0.08 ± 0.01%/day and 0.23 ± 0.02%/day, respectively.

### 3.2. Nutrient Levels in the Rare Minnow

The crude protein percentage of fish in the treatment group increased at 15 and 30 days and decreased at 45 and 60 days compared to the control fish. Meanwhile, the ether extract content of fish in the treatment group decreased and then increased with time. The overall trend of ash content of the whole fish supplemented with ZnO NPs in the diets was increased, but there was no significant change in moisture content (*p* > 0.05, Table 2).

### 3.3. Zn Content in the Muscle Tissue of Rare Minnow and Diets

Dietary supplementation with ZnO NPs altered the Zn content in the muscle tissues of rare minnow and diets. Zn contents of control fish were ranged from 15.01 to 18.21 µg/g. For the ZnO NPs fed groups, the Zn contents were significantly increased in comparison with the control levels (*p* < 0.05, Table 3).

### 3.4. Blood Analysis of Rare Minnow

**Figure 1:**
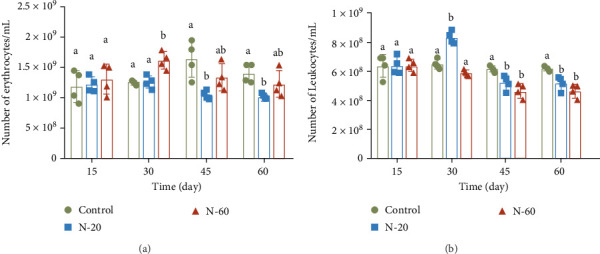
Effects of dietary supplement ZnO NPs at 0, 20, and 60 mg/kg on the number of erythrocytes and leukocytes in blood: (a) erythrocytes and (b) leukocytes. Data are presented as mean ± standard deviation. Divergent letters within the same time period represent remarkable statistical differences between the treatment and control groups (*p* < 0.05).

Dietary supplement of ZnO NPs altered the number of erythrocytes and leukocytes in the blood. Erythrocyte counts increased in the treated groups at 15 and 30 days and decreased at 45 and 60 days than in the control group, with remarkable decreases in the N-20 group as 1.1 × 10^9^ ± 6.1 × 10^7^ and 1.0 × 10^9^ ± 3.7 × 10^7^ (*p* < 0.05, Figure 1a). The leukocyte counts in the treatment groups changed in line with the erythrocyte counts, with a significant decrease starting at 45 days in the N-20 group and at 30 days in the N-60 group (*p* < 0.05, Figure 1b).

### 3.5. Hepatic Histological Indices of Rare Minnow

**Figure 2:**
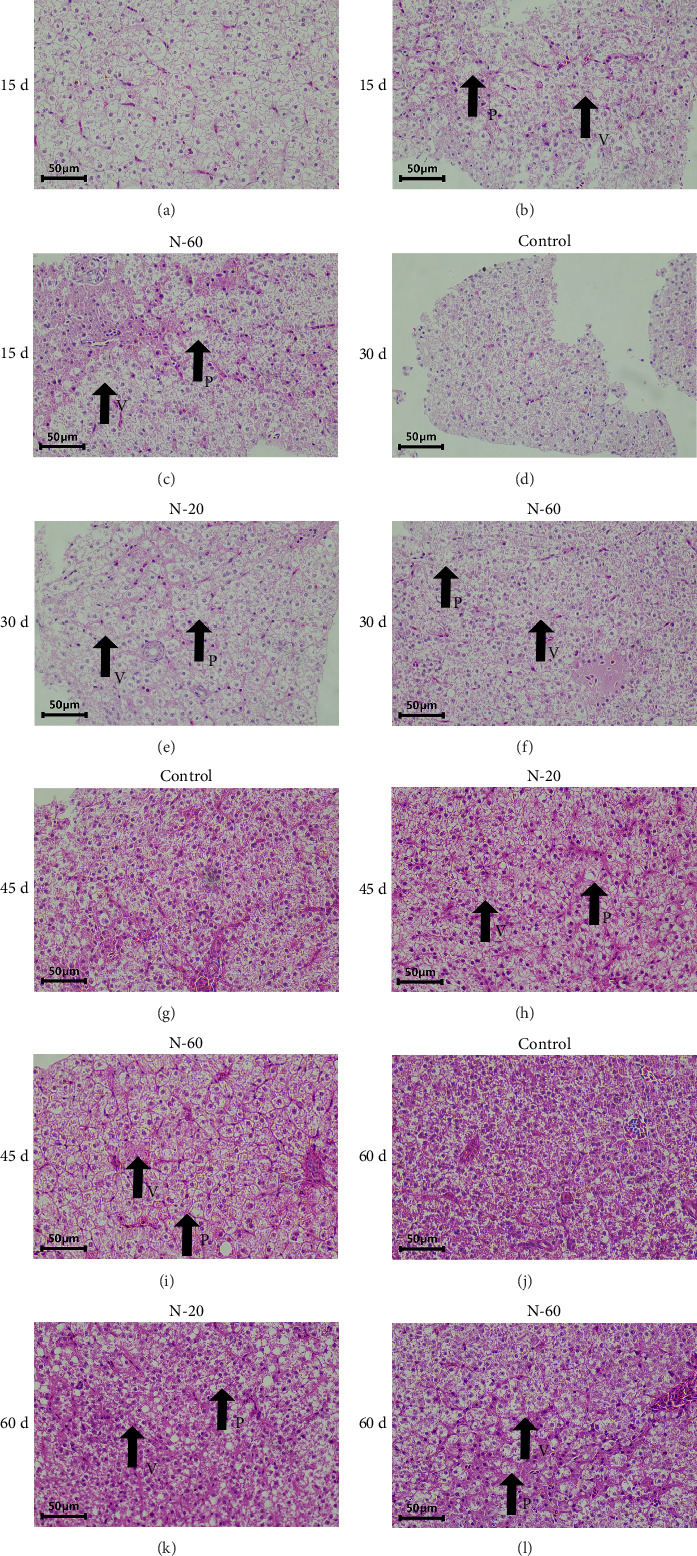
Effects of hepatic tissues of rare minnow after dietary supplement of 0, 20, and 60 mg/kg ZnO NPs (*n* = 3). (a, d, g, j) Normal hepatic tissues (fed with the control diet for 15, 30, 45, and 60 days, respectively). (b, e, h, k) Hepatic tissues of 20 mg/kg ZnO NPs-fed group (fed with the N−20 diet for 15, 30, 45, and 60 days, respectively). (c, f, i, l) Hepatic tissues of 60 mg/kg ZnO NPs-fed group (fed with the N−20 diet for 15, 30, 45, and 60 days, respectively). *P* = hepatocyte vacuolization; *V* = absence of nucleus.

Control fish showed normal hepatocyte structure, characterized by large nuclei stained dark blue-violet, centrally located in hepatocytes, fewer and more visible hepatic blood sinusoids, and clear red blood cells in microvessels (Figure 2a,d,g,j). However, the N-20 and N-60 groups showed pathological features, including severe vacuolization of hepatocytes, loss of hepatocyte shape, swelling of hepatocytes, and displacement of nuclei in some cells (Figure 2b,c,e,f,h,i,k,l). The liver injury status index showed a significant increase in the treatment groups (*p* < 0.05, Table 4).

### 3.6. Relative Expression and Primer Amplification Efficiency of Muscle Growth-Related Genes of Rare Minnow

**Figure 3:**
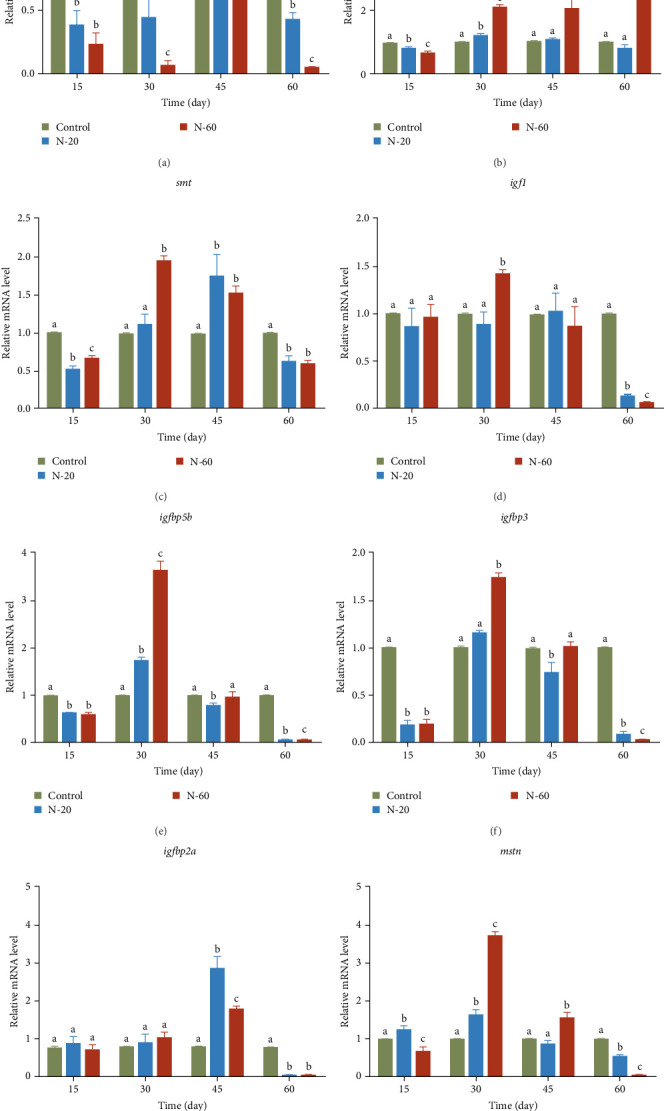
Effects of dietary supplement ZnO NPs at 0 mg/kg, 20 mg/kg, and 60 mg/kg on muscle tissue growth-related genes (*gh* (a), *ghrb* (b), *smt* (c), *igf1* (d), *igfbp5b* (e), *igfbp3* (f), *igfbp2a* (g), and *mstn* (h)) of rare minnow. Data are standardized to *β*-actin and expressed as mean ± standard deviation. Divergent letters within the same time period represent remarkable statistical differences between the treatment and control groups (*p* < 0.05).

To further investigate the impacts of dietary ZnO NPs on the growth performance of rare minnow, the researchers measured the mRNA expression levels of growth-related genes in the muscles of rare minnows (Figure 3). The expression level of *gh* of N-20 and N-60 was remarkably decreased compared to the control during the 60-day experimental period (*p* < 0.05, Figure 3a). At 15 days, the expression level of *ghrb*, *smt*, *igf1*, *igfbp5b*, and *igfbp3* genes was remarkably reduced in the treated group in comparison to the control group (*p* < 0.05, Figure 3b–f). At 30 days, the mRNA expression levels of all genes in the experimental group were remarkably upregulated compared to the control group except for the *gh* gene (Figure 3a–h). At 60 days, the mRNA expression levels of all genes in the experimental group were remarkably decreased compared to the control group (*p* < 0.05, Figure 3a–h), except for the *ghrb* gene of N-60. The statistical analysis showed that adding ZnO NPs to the diet negatively affected muscle growth. Primer amplification efficiencies for eight genes related to muscle growth and *β-actin* in rare minnow ranged from 96.55% to 98.66% (Table 5).

### 3.7. Relative Expression and Primer Amplification Efficiency of Liver Immunity-Related Genes of Rare Minnow

**Figure 4:**
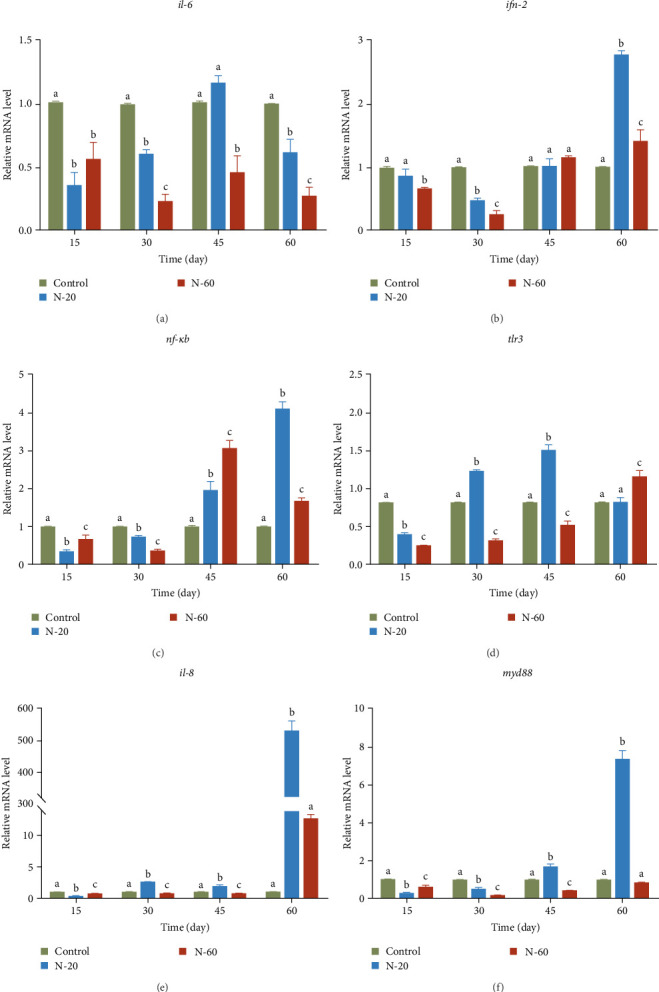
Effects of dietary supplement ZnO NPs at 0 mg/kg, 20 mg/kg, and 60 mg/kg on liver immunity-related genes (*il-6* (a), *ifn-2* (b), *nf-кb* (c), *tlr3* (d), *il-8* (e), and *myd88* (f)) of rare minnow. Data are standardized to *β*-actin and expressed as mean ± standard deviation. Divergent letters within the same time period represent remarkable statistical differences between the treatment and control groups (*p* < 0.05).

To examine further the effect of dietary ZnO NPs on the immune function of 3-month-old rare minnow, the researchers measured the mRNA expression levels of immunomodulation-related genes in the liver of rare minnow (Figure 4). During the 60-day test cycle, *il-6* mRNA expression levels were significantly reduced in the treatment groups compared to the control group, except for the N-20 group at 45 days (*p* < 0.05, Figure 4a). In comparison with the control group, the mRNA expression levels of the *ifn-2* and *nf-κb* genes in the experimental group significantly decreased at 15–30 days, followed by a significant increase at 45–60 days (*p* < 0.05, Figure 4b,c). In comparison with the control group, the mRNA expression levels of *tlr3* and *il-8* genes in the N-20 group decreased remarkably at 15 days and increased significantly at 30–60 days (*p* < 0.05, Figure 4d,e). The N-60 group significantly increased only at 60 days (*p* < 0.05, Figure 4d,e). During the test cycle, *myd88* mRNA expression levels were lower than those of the control in all treatment groups except for the N-20 group at 45 and 60 days (*p* < 0.05, Figure 4f). Primer amplification efficiencies for six genes related to liver immunity and *β-actin* in the rare minnow ranged from 97.33% to 98.57% (Table 6).

## 4. Discussion

As a crucial nanomaterial, the application of ZnO NPs in animal feed has received considerable attention in recent years [24] and has become one of the hot spots of current research [25]. The results of many experiments showed that fish weight, weight gain, and specific growth rates were significantly higher in fish supplemented with ZnO NPs compared to other forms of Zn (e.g., bulk ZnO) [26]. In this experiment, we tested the impacts of dietary supplementation with 0, 20 and 60 mg/kg of ZnO NPs on the growth performance of rare minnow. The results indicated that the supplementation of ZnO NPs in the diet improved the body length of the rare chevron fish at 15 days, CF at 45 and 60 days and SGR from 30 to 60 days. The effect of ZnO NPs was most pronounced when 60 mg/kg of ZnO NPs was added to the diet. This is in agreement with the findings of Mahboub et al. [27].

Dietary addition of micronutrients, such as Zn, can influence fish growth performance and other biomarkers [28]. Diets supplemented with moderate amounts of Zn promote growth performance of aquatic organisms, while different chemical forms of Zn influence bioavailability and production efficiency in aquatic organisms [29]. A certain amount of ZnO NPs in a short period of time can be safely added to aquatic animal feeds to treat Zn deficiency and promote aquatic animal growth. However, during the experimental period of 60 days, the weight of the fish in the treated group decreased and at 15 days, and the SGR of the fish in the treated group decreased significantly. This is consistent with the findings of Rashidian et al. [30]. ZnO NPs may compete with other nutrients in the gut, thus reducing their absorption [31], which ultimately affects fish growth and body weight. Generally, ZnO NPs are absorbed mainly as Zn and partly enter the body in the form of particles, which can increase Zn levels in tissues [32]. Therefore, weight loss in rare minnow may be related to elevated Zn levels caused by dietary ZnO NPs.

Fish growth is closely related to liver function. As a major metabolic organ, the liver is involved in the synthesis, storage, and regulation of nutrients, especially in the metabolism of proteins and sugars [33]. The health status of the liver directly affects the growth rate and weight gain of fish. In rare minnow, dietary supplementation with ZnO NPs resulted in liver injury with a significant increase in the liver injury status index, as evidenced by extensive cytoplasmic vacuolization, focal necrosis, and irregular or missing nuclei, which is in agreement with the results of the study by Su et al. [34]. Similar phenomena were observed in gilthead seabream (*Sparus aurata*) [35] and Nile tilapia [36].

Prolonged high doses of ZnO NPs lead to altered growth hormone (GH) synthesis [18]. ZnO NPs were discovered to regulate muscle tissue mitosis and cell specification via affecting GH and insulin-like growth factor 1 (IGF-1) [26]. It has been reported that hormonal alterations in aquatic organisms can be induced at the cellular and molecular levels under ZnO NPs treatment. And at the molecular level, the transcript levels of *gh*, *igf-i*, *insulin*, and *ira* genes were significantly downregulated [18]. GH is a peptide hormone secreted by the pituitary gland, which promotes cellular growth, renewal, and reproduction both humans and animals. It has numerous targets and varied actions in vertebrates and primarily functions as a growth stimulator and influencing metabolic processes [37]. According to reports, fish growth hormone can alter fish behavior by improving appetite, aggression, and swimming ability, while decreasing antipredator behavior [38]. IGF1 promotes growth, maturation, and specification of fish and other aquatic animals [39]. GH and IGF1 are important growth markers in fish [40]. IGFbp5b, IGFbp2a, and IGFbp3 encode proteins that bind and transport IGF1, playing a crucial role in muscle growth [41]. Therefore, the long-term feeding of feed supplemented with ZnO NPs in this experiment. Due to the decreased expression levels of growth genes such as *gh*, *igf1*, and *igfbp5b* as a result of high levels of accumulated Zn in the body, the above functions may be altered, thereby affecting the behavior and food intake of rare minnow.

Hematological analysis is one of the most valuable indicators to evaluate health and physiological alterations in fish [42]. The findings of this study demonstrated that the addition of ZnO NPs increased the number of red and white blood cells in the blood in the first period, but decreased in the later period of treatment. Zn is considered to be a vital component of the immune system, which is necessary for the intactness of the immune organs [43]. It has been reported that when nanoparticles enter the organism, they can engage with immunity cells to induce an inflammatory reaction, associated with the production of signal molecules (cytokines), which allow interaction between immune cells and regulate molecular events [44]. The immune response of fish can be assessed by measuring immunomodulatory genes. TLR3, IL-6, IFN-2, IL-8, NF-*κ*B, and MyD88 play key roles in the regulation of inflammatory and immune processes. The toll-like receptor (TLR) family plays a key role in the defense against conditions like immune inflammation and cancer. Activation of TLR3 causes the production of a variety of inflammatory cytokines and chemotactic factors, which mobilize and activate immune cells to participate in antiviral responses [45]. The death region of MyD88 upregulates subsequent signal molecules like IL-1 and IL-4 receptor-associated kinase, tumor necrosis factor receptor-associated factor 6, transforming growth factor *β*1, which engages NF-*κ*B, and P38 mitogen-activated protein kinase and activator protein 1, resulting in the generation of proinflammatory cytokines [46]. Previous studies have shown that activation of the TLR signal pathway upregulates the expression of *tlr3*, *myd88*, and the subsequent proinflammatory cytokines *il-6*, *il-8*, and *ifn-2* [47, 48]. The findings of this study revealed that the addition of ZnO NPs decreased the gene mRNA expressed level of *il-6* and downregulated the gene mRNA expressed levels of proinflammatory factors (*tlr3*, *ifn-2*, *il-8*, *nf-кb*, and *myd88*) in the preexperimental period compared to the control group. However, the gene expression levels of proinflammatory factors were significantly upregulated later in the experiment. This may be the result of the positive effect of short duration of ZnO NPs on immune function in the early stage, but prolonged use may have negative effects on immunity. The liver not only plays an important role in metabolism but is also a key component of the immune system [49, 50]. Liver injury may lead to a decrease in immune function [49, 50], which in turn reduces the resistance of fish to pathogens and further affects their growth performance.

## 5. Conclusion

As feed supplement, ZnO NPs negatively affected the body weight of rare minnow, with a trend of increasing and then decreasing body length, but decreasing and then increasing CF and SGR. Additionally, the accumulated Zn level was significantly higher (*p*  < 0.05), and the liver injury index was significantly higher (*p*  < 0.05) in the dietary ZnO NPs group compared to the control group. The number of erythrocytes and the number of leukocytes in the blood of rare minnow increased and then decreased after treatment with ZnO NPs. It was further found that accumulation of ZnO NPs in muscle tissue for 60 days as a feed additive significantly decreased the mRNA expressed levels of growth-related genes (*gh*, *igf1*, *igfbp5b*, *igfbp2a*, *igfbp3*, and *smt*), and accumulation in liver tissue for 60 days significantly increased the mRNA expressed levels of immune-modulation-related genes (*inf-2*, *tlr3*, *il-8*, *myd88*, and *nf-κb*) (*p* < 0.05).

## Figures and Tables

**Table 1:** Growth performance in the rare minnow after dietary supplementation with different levels of ZnO NPs.

Items	Body weight (g)	Body length (mm)	CF (%)	SGR (%)
15 days control	0.39 ± 0.07^a^	29.13 ± 1.06^a^	1.59 ± 0.17^a^	0.65 ± 0.02^a^
15 days N-20	0.37 ± 0.02^a^	29.38 ± 0.82^a^	1.47 ± 0.13^a^	0.45 ± 0.02^b^
15 days N-60	0.36 ± 0.08^a^	29.40 ± 1.24^a^	1.43 ± 0.18^a^	0.19 ± 0.01^c^
30 days control	0.39 ± 0.05^a^	29.62 ± 1.40^a^	1.52 ± 0.22^a^	0.01 ± 0.00^a^
30 days N-20	0.38 ± 0.07^a^	29.54 ± 1.45^a^	1.49 ± 0.20^a^	0.02 ± 0.00^b^
30 days N-60	0.36 ± 0.03^a^	29.48 ± 1.31^a^	1.42 ± 0.20^a^	0.02 ± 0.00^b^
45 days control	0.39 ± 0.05^a^	30.18 ± 1.41^a^	1.44 ± 0.20^a^	0.03 ± 0.00^a^
45 days N-20	0.38 ± 0.07^a^	29.59 ± 1.77^a^	1.50 ± 0.26^a^	0.08 ± 0.01^b^
45 days N-60	0.37 ± 0.05^a^	29.53 ± 0.93^a^	1.44 ± 0.12^a^	0.23 ± 0.02^c^
60 days control	0.42 ± 0.06^a^	30.57 ± 1.70^a^	1.50 ± 0.25^a^	0.65 ± 0.10^a^
60 days N-20	0.42 ± 0.04^a^	30.47 ± 1.45^a^	1.51 ± 0.24^a^	0.69 ± 0.06^a^
60 days N-60	0.42 ± 0.04^a^	30.42 ± 0.62^a^	1.50 ± 0.09^a^	0.76 ± 0.08^a^

*Note:* Data are measured as mean ± standard deviation. Divergent letters within the same time period represent significant statistical differences between the treatment and control groups (*p* < 0.05).

Abbreviations: CF, condition factor; SGR, specific growth rate.

**Table 2:** Nutrient levels in rare minnow after dietary supplementation with different levels of ZnO NPs.

Items	Crude protein (%)	Ether extract (%)	Ash (%)	Moisture (%)
15 days control	62.01 ± 1.44^a^	20.58 ± 0.80^a^	11.68 ± 1.05^a^	70.64 ± 0.11^a^
15 days N-20	65.55 ± 2.81^ab^	20.00 ± 0.60^a^	12.33 ± 0.99^a^	72.35 ± 1.43^a^
15 days N-60	71.67 ± 1.88^b^	16.77 ± 1.14^b^	14.41 ± 0.69^a^	72.20 ± 1.20^a^
30 days control	68.70 ± 1.02^a^	15.42 ± 1.94^a^	13.07 ± 0.40^ab^	75.12 ± 1.83^a^
30 days N-20	69.06 ± 1.55^a^	18.52 ± 1.49^a^	11.81 ± 0.34^a^	73.89 ± 1.04^a^
30 days N-60	70.04 ± 2.23^a^	13.31 ± 1.94^b^	14.16 ± 0.95^b^	73.94 ± 1.70^a^
45 days control	73.31 ± 2.21^a^	8.98 ± 0.35^a^	15.40 ± 0.52^a^	76.45 ± 1.45^a^
45 days N-20	67.11 ± 2.72^a^	13.98 ± 1.29^b^	17.97 ± 0.25^b^	74.69 ± 2.05^a^
45 days N-60	67.87 ± 2.29^a^	11.94 ± 1.39^ab^	10.58 ± 0.81^c^	74.35 ± 0.92^a^
60 days control	71.39 ± 2.80^a^	8.73 ± 1.78^a^	11.78 ± 1.12^a^	77.04 ± 1.77^a^
60 days N-20	68.63 ± 2.85^a^	11.51 ± 1.79^a^	16.86 ± 0.72^b^	74.65 ± 1.91^a^
60 days N-60	63.53 ± 2.14^a^	12.15 ± 1.15^a^	13.67 ± 1.23^ab^	75.88 ± 1.83^a^

*Note:* Data are measured as mean ± standard deviation. Divergent letters within the same time period represent remarkable statistical differences between the treatment and control groups (*p* < 0.05).

**Table 3:** Zn content in muscle tissues of the rare minnow after dietary supplementation with different levels of ZnO NPs and actual measurements of Zn content in the diets of each treatment group.

Samples		The concentration of Zn (µg/g dry weight)		
		The control group	The N-20 group	The N-60 group
Muscle tissues	15 days	15.01 ± 0.08^a^	17.83 ± 0.42^b^	19.12 ± 0.47^c^
	30 days	17.68 ± 0.24^a^	21.68 ± 0.47^b^	26.51 ± 0.41^c^
	45 days	18.21 ± 0.43^a^	27.51 ± 0.71^b^	31.25 ± 0.82^c^
	60 days	18.00 ± 0.12^a^	30.87 ± 0.41^b^	35.71 ± 0.41^c^

Treatment group diets (actual measurements)		66.62 ± 1.46^a^	78.39 ± 1.78^b^	84.89 ± 1.48^c^

*Note:* Data are measured as mean ± standard deviation. Divergent letters within the same time period represent remarkable statistical differences between the treatment and control groups (*p* < 0.05).

**Table 4:** The liver injury status index of rare minnow supplemented with 0, 20, and 60 mg/kg ZnO NPs in diets for 15, 30, 45, and 60 days.

Feeding days (days)	*I*_*h*_		
	Control group	N-20 group	N-60 group
15	6.67 ± 0.47^a^	7.67 ± 0.47^b^	10.67 ± 0.47^c^
30	6.00 ± 0.82^a^	9.00 ± 0.82^b^	12.33 ± 0.94^c^
45	5.33 ± 0.47^a^	15.67 ± 0.47^b^	19.00 ± 0.82^c^
60	5.67 ± 0.47^a^	19.33 ± 0.47^b^	21.67 ± 0.47^c^

*Note:* Weight values ranged between 1 and 3 (most severe), and scores ranged from 0 (no features/alterations observed) to 6 (diffuse). *w* = 1: intercellular edema and structural alterations, *w* = 2: nuclear alterations and atrophy, *w* = 3: necrosis and vacuolar degeneration. Different letters in the same column indicate significant differences (*p* < 0.05). Values are measured as mean ± standard deviation.

**Table 5:** Primer amplification efficiency of growth-related genes.

Gene	Gene description	Amplification efficiency (%)
*gh*	Growth hormone	98.33
*ghrb*	Growth hormone receptor b	97.58
*smt*	Somatostatin	97.01
*igf1*	Insulin-like growth factor 1	97.59
*igfbp5b*	Insulin-like growth factor binding-protein 5	96.55
*igfbp3*	Insulin-like growth factor binding protein 3	98.15
*igfbp2a*	Insulin-like growth factor binding protein 2a	98.66
*mstn*	Myostatin	98.31
*β-actin*	Beta-Actin	98.01

**Table 6:** Primer amplification efficiency of immunity-related genes.

Gene	Gene description	Amplification efficiency (%)
*il-6*	Interleukin 6	97.83
*ifn-2*	Inverted formin 2	98.57
*nf-кb*	Nuclear factor-kappa B	97.48
*tlr3*	Toll-like receptor 3	98.05
*il-8*	Interleukin-8	97.86
*myd88*	Myeloid differentiation factor 88	97.33
*β-actin*	Beta-Actin	98.01

## Data Availability

The data that support the findings of this study are available on request from the corresponding author. The data are not publicly available due to privacy or ethical restrictions.

## Supporting Information

Supporting Information Ingredient compositions of the basal diet of rare minnow and real-time quantitative PCR primer sequences are shown in Supporting Information.
