# Supplementary material for: The Effect of Dietary Zinc Oxide Nanoparticles on Growth Performance, Zinc in Tissues, and Immune Response in the Rare Minnow (Gobiocypris rarus)
Source: Aquac Nutr. 2024 Dec 2;2024:9553278. doi: 10.1155/anu/9553278 (PMC11628176; doi:10.1155/anu/9553278)
Supplement: Supporting Information — Ingredient compositions of the basal diet of rare minnow and real-time quantitative PCR primer sequences are shown in Supporting Information. [file 9553278.f1.docx]

**The effect of dietary zinc oxide nanoparticles on growth performance, zinc in tissues and immune response in the rare minnow (*Gobiocypris rarus*)**

**Huanhuan Li ^a^, Menghan Wu^c,d^, Jinming Wu^b^, Jing Wan^a^, Yongfeng He^c^,** **Yifan Ding ^a^ , Jun Liu^a, *^, Liangxia Su^a, *^**

*^a^ Hubei Key Laboratory of Animal Nutrition and Feed Science, Engineering Research Center of Feed Protein Resources on Agricultural By-Products, Ministry of Education,* *Wuhan Polytechnic University, Wuhan, China*

*^b^Key Laboratory of Freshwater Biodiversity Conservation, Ministry of Agriculture and Rural Aﬀairs of China,**Chinese Academy of Fishery Sciences Yangtze River Fisheries Research Institute, Wuhan, China*

*^c^ The Key Laboratory of Aquatic Biodiversity and Conservation of Chinese Academy of Sciences, Institute of Hydrobiology Chinese Academy of Sciences, Wuhan, Hubei, China*

*^d^ University of Chinese Academy of Sciences, Beijing, China*

^*^ Correspondence to: Jun Liu, Hubei Key Laboratory of Animal Nutrition and Feed Science, Engineering Research Center of Feed Protein Resources on Agricultural By-Products, Ministry of Education, Wuhan Polytechnic University, Wuhan, China; E-mail: [673164434@qq.com](mailto:673164434@qq.com)

**^**^**Correspondence to: Liangxia Su, Hubei Key Laboratory of Animal Nutrition and Feed Science, Engineering Research Center of Feed Protein Resources on Agricultural By-Products, Ministry of Education, Wuhan Polytechnic University, Wuhan, China; E-mail: suliangxia027@126.com

**Supplementary Table**

**SI Table S1. Ingredient compositions in basal diet of rare minnows.**

| Items | Composition |
| --- | --- |
| Ingredients | Content (%) |
| Fish meal | 48.0 |
| Dextrin | 16.5 |
| Mineral mixtures | 2.0 |
| Soybean oil | 2.5 |
| Vitamin blends | 1.0 |
| Cellulose | 26.5 |
| Spirulina powder | 2.0 |
| Binders | 1.5 |
| Total | 100 |

Note: Each kilogram of the mineral mixture described contains (g): 10.0 g NaCl, 163.11 g MgSO_4_•7H_2_O, 250 g NaH_2_PO_4_•2H_2_O, 320 g KH_2_PO_4_, 153.01 g Ca(H_2_PO_4_)_2_•2H_2_O, 45.72 g FeSO_4_, 35 g C_6_H_10_CaO_6_•5H_2_O, 3.56 g ZnSO4•7H_2_O, 1.23 g MnSO_4_•4H_2_O, 0.31 g CuSO_4_•5H_2_O, 0.69 g CoSO_4_•6H_2_O, 2.30 g KI, 15.07 g starch. Each kilogram of the vitamin premix contains: 16 g thiamin, 16 g riboflavin, 16 g pyridoxine, 0.02 g cyanocobalamin, 40 g pantothenic acid, 4 g folic acid, 80 g inositol, 80 g niacin, 0.08 g biotin, 80 g ascorbic, 88 g vitamin A, 16 g Vitamin D, 40 g Vitamin E, 8 g Vitamin K, 515.9 g starch.

**SI Table S2.** **Nutrient composition in basal diet of rare minnow**

| Items | Measured Content | | |
| --- | --- | --- | --- |
|  | The control group | The N-20 group | The N-60 group |
| Crude protein (%) | 39.96 ± 0.46^a^ | 39.24 ± 0.47^a^ | 39.30 ± 0.49^a^ |
| Ether extract (%) | 7.08 ± 0.17^a^ | 7.00 ± 0.72^a^ | 7.21 ± 0.32^a^ |
| Ash (%) | 6.348 ± 0.73^a^ | 6.94 ± 0.09^a^ | 6.66 ± 0.47^a^ |
| Moisture (%) | 8.85 ± 0.11^a^ | 8.43 ± 0.38^a^ | 8.39 ± 0.11^a^ |

Data are measured as mean ± standard deviation. Different letters indicate a statistically significant difference in the treated group compared to the control group (*P* < 0.05).

**SI Table S3. Sequences of the primers for quantitative real-time PCR**

| Gene | Gene description | Primer sequence (5' to 3') |
| --- | --- | --- |
| *gh* | Growth hormone | F: ACCAGCCAGCACCTCTTA |
|  |  | R: ACACCTCTGCAAACTCCC |
| *smt* | Somatostatin | F: ATTCCAGCCTATCCCTCC |
|  |  | R: CATAAATGACTAGCGTGTTCC |
| *mstn* | Myostatin | F: GCCAAGGGTGATGTGACGA |
|  |  | R: TGTGGTCCAGTGGGTAAT |
| *igf1* | Insulin like growth factor 1 | F: ATTGTGGACGAATGCTGC |
|  |  | R: TTCTTCTGATGAACCTCCTTAC |
| *igfbp5b* | Insulin-like growth factor binding-protein 5 | F: TGCGGCTGCTGCTTGACT |
|  |  | R: AGGTGGGTGCGGTGGTTT |
| *igfbp2a* | Insulin-like growth factor binding protein 2a | F: CGGGAGATGTGGACGAAA |
|  |  | R: CTGGCGGTGATGAAGGAC |
| *igfbp3* | Insulin like growth factor binding protein 3 | F: TTTCCCGTCTCCTCCCTT |
|  |  | R: GGCAGAGCCATCCAATCA |
| *ghrb* | Growth hormone receptor b | F: AGTGTTGCCTGGTGTTGATG |
|  |  | R: CTCCACGTTGAAAGACAT |
| *tlr3* | Toll-like receptor 3 | F: GACTTCACCAGGTAATGGAGGAC |
|  |  | R: AAGGTTTCAACATGCCACGAC |
| *il-6* | Interleukin 6 | F: CAAAAAGCGTGTTTTCAGCA |
|  |  | R: GGCATGACGTGTTCACAAAG |
| *ifn-2* | Inverted formin 2 | F: ACAGTCAAGCAGGAGGAGGA |
|  |  | R: TCACTGGCGCTGTCTGTATC |
| *il-8* | Interleukin-8 | F: AGGAATGAGTCTTCGAGGTGTG |
|  |  | R: GTGATTTGTTGGCAAGGATCTT |

| *nf-кb* | Nuclear factor-kappa B | F: AACTCAGTCAGGCTCCATTGC  R: GACAGTGCTCTCCGTCTTTCC |
| --- | --- | --- |

| *myd88* | Myeloiddifferentiationfactor88 | F: GGTGGTAATTTCCGATGA |
| --- | --- | --- |
|  |  | R: GTAGACAACAGGGATAAGG |
| *β-actin* | Beta-actin | F: ACCGCAAGATTCCATAC |
|  |  | R: GTCCGTGACATCAAAGAG |
